# Supplementary figures and images for: The invasion pore induced by Toxoplasma gondii
Source: EMBO Rep. 2025 Sep 19;26(20):5009–26. doi: 10.1038/s44319-025-00565-8 (PMC12549902; doi:10.1038/s44319-025-00565-8)

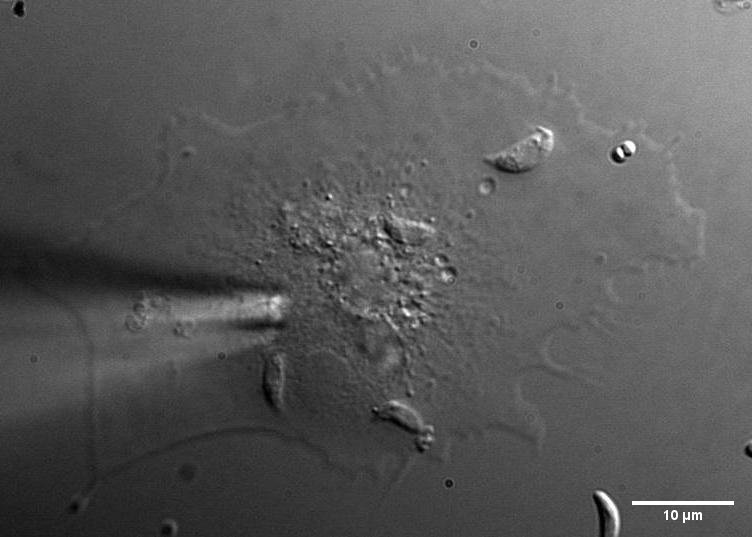

Supplement: Supplementary file 2 — Source data Fig. 1 [file 44319_2025_565_MOESM2_ESM.zip › Figure 1/Figure 1a/Figure 1 A.jpg]

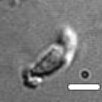

Supplement: Supplementary file 2 — Source data Fig. 1 [file 44319_2025_565_MOESM2_ESM.zip › Figure 1/Figure 1a/P1.jpg]

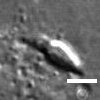

Supplement: Supplementary file 2 — Source data Fig. 1 [file 44319_2025_565_MOESM2_ESM.zip › Figure 1/Figure 1a/P2.jpg]

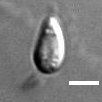

Supplement: Supplementary file 2 — Source data Fig. 1 [file 44319_2025_565_MOESM2_ESM.zip › Figure 1/Figure 1a/P3.jpg]

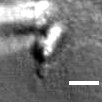

Supplement: Supplementary file 2 — Source data Fig. 1 [file 44319_2025_565_MOESM2_ESM.zip › Figure 1/Figure 1a/P4.jpg]
