# Supplementary material for: The invasion pore induced by Toxoplasma gondii
Source: EMBO Rep. 2025 Sep 19;26(20):5009–26. doi: 10.1038/s44319-025-00565-8 (PMC12549902; doi:10.1038/s44319-025-00565-8)
Supplement: Supplementary file 7 — Expanded View Figures [file 44319_2025_565_MOESM7_ESM.pdf]

Expanded View Figures

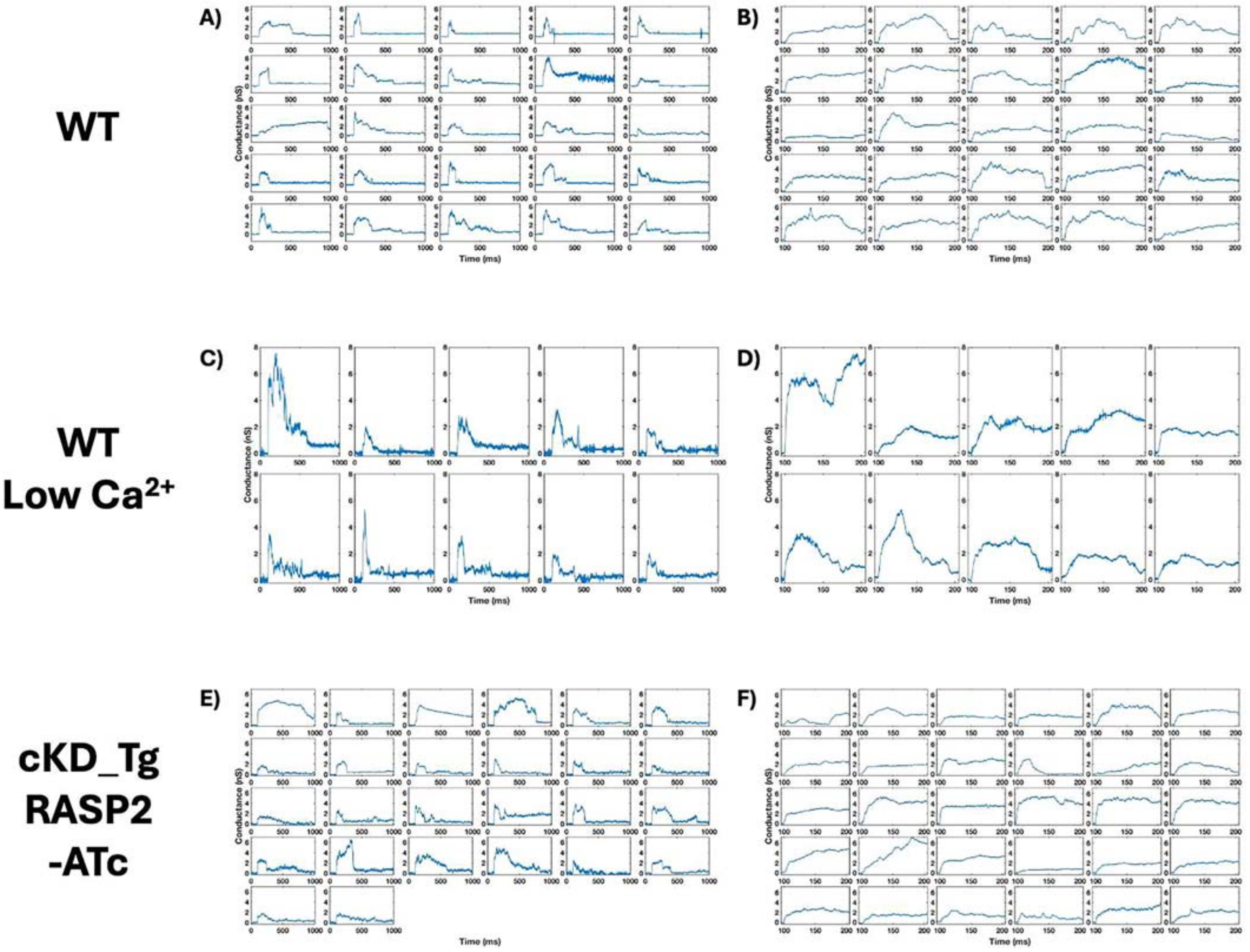

Figure EV1. Galleries of conductance transients induced by WT and cKD_TgRASP2 -ATc parasites.

(**A**) Gallery of 25 WT parasite conductance transients calculated from recorded current measured using −60 mV holding potential in an external buffer containing 2.0 mM $CaCl_2$. The initial 100 ms of baseline is plotted prior to the detection of the transient. (**B**) Gallery of 25 WT parasite conductance transients calculated from recorded current measured using −60 mV holding potential in an external buffer containing 2.0 mM $CaCl_2$. The initial 5 ms of baseline prior to the detection of the transient and the initial 105 ms of each recorded transient are plotted. (**C**) Gallery of ten WT parasite conductance transients in low external calcium, calculated from recorded current measured using −60 mV holding potential in an external buffer containing 0.1 mM $CaCl_2$. The initial 100 ms of baseline is plotted prior to the detection of the transient. (**D**) Gallery of 10 WT parasite conductance transients in low external calcium, calculated from recorded current measured using −60 mV holding potential in an external buffer containing 0.1 mM $CaCl_2$. The initial 5 ms of baseline prior to the detection of the transient and the initial 105 ms of each recorded transient are plotted. (**E**) Gallery of 26 cKD_TgRASP2 -ATc parasite conductance transients calculated from recorded current measured using −60 mV holding potential in an external buffer containing 2.0 mM $CaCl_2$. The initial 100 ms of baseline is plotted prior to the detection of the transient. Because no ATc or solvent is applied, aside from the genetic alteration of the parasite, no change in the protein complement of this parasite line compared to WT is expected. (**F**) Gallery of 26 cKD_TgRASP2 -ATc conductance transients calculated from recorded current measured using −60 mV holding potential, showing the initial 105 ms of the transients. 5 ms of baseline is included prior to detection of the transient. Because no ATc or solvent is applied, aside from the genetic alteration of the parasite, no change in the protein complement of this parasite line compared to WT is expected.

 This is a U.S. Government work and not under copyright protection in the US; foreign copyright protection may apply

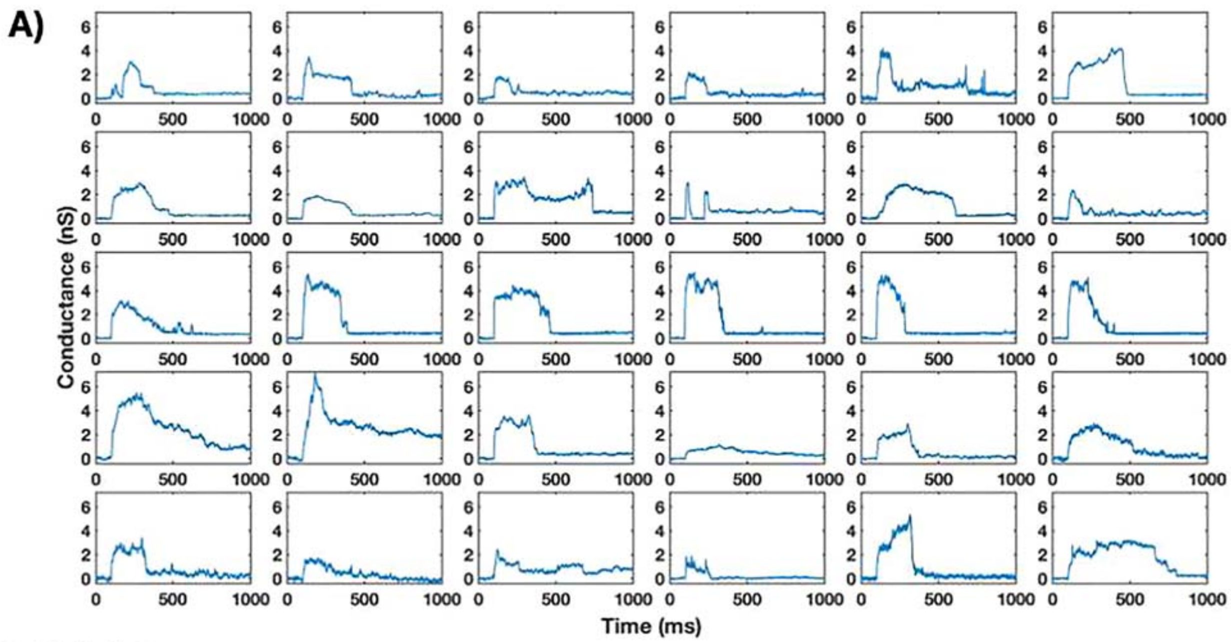

KD RON2

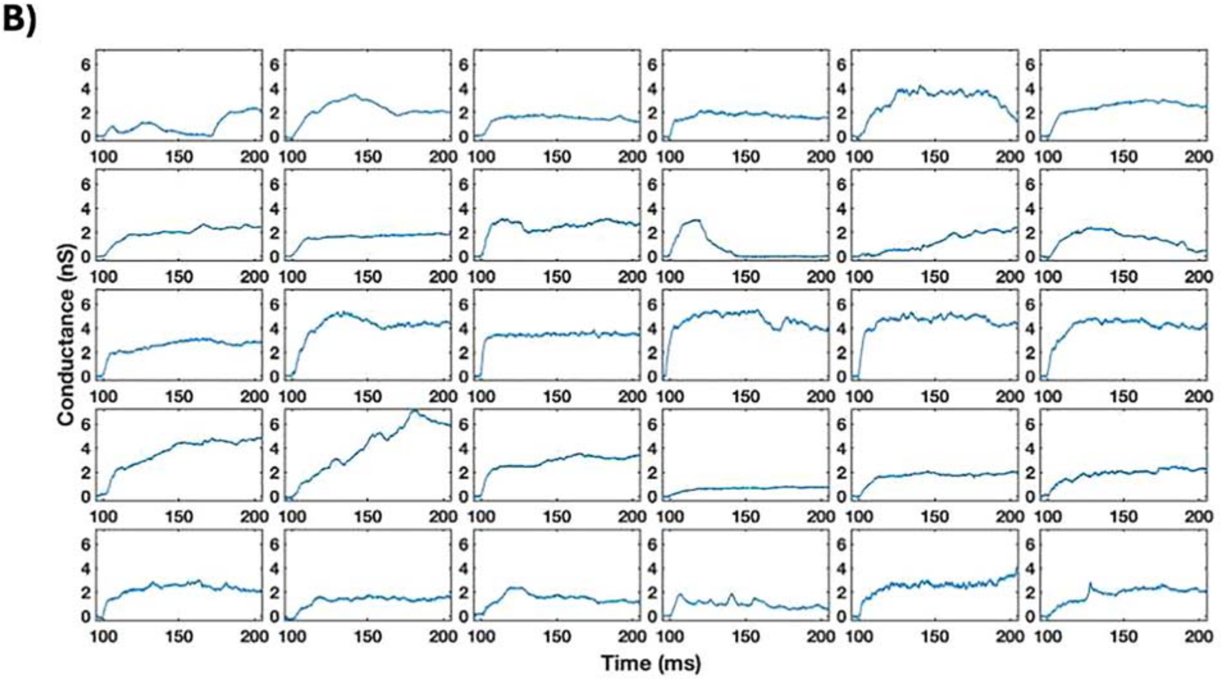

Figure EV2. Galleries of conductance transients induced by KD-RON2 parasites.

(**A**) Gallery of 30 KD-RON2 parasite conductance transients calculated from recorded current measured using −60 mV holding potential in an external buffer containing 2.0 mM $CaCl_2$. The initial 100 ms of baseline is plotted prior to the detection of the transient. (**B**) Gallery of 30 KD-RON2 parasite conductance transients calculated from recorded current measured using −60 mV holding potential, showing the initial 105 ms of the transients in an external buffer containing 2.0 mM $CaCl_2$. The initial 5 ms of baseline is plotted prior to the detection of the transient.

This is a U.S. Government work and not under copyright protection in the US; foreign copyright protection may apply

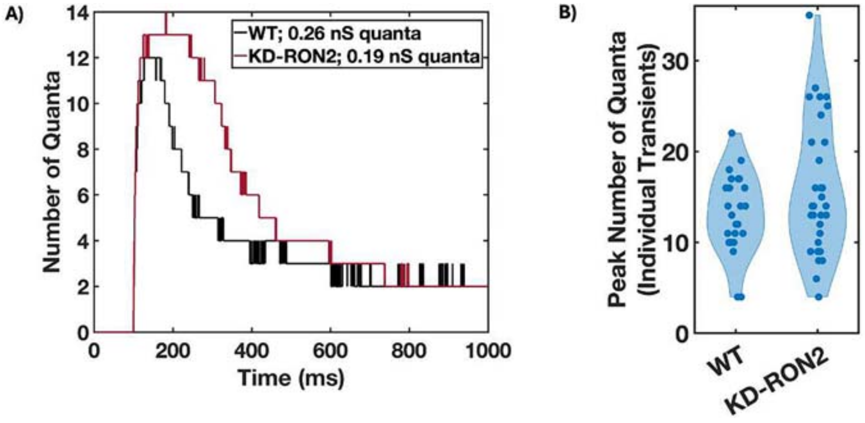

Figure EV3. The same number of quantal units contributes to the conductance maxima of WT and KD-RON2 transients.

(**A**) Transformation of the mean transient conductance (Fig. 3A) using the peak quantal sizes for WT (0.26 nS) and KD-RON2 (0.19 nS), rounded to the nearest integer. (**B**) Violin plots of the number of quantal units at the maximum conductance of WT and KD-RON2 transients ($n = 25$ and 30, respectively).

 This is a U.S. Government work and not under copyright protection in the US; foreign copyright protection may apply

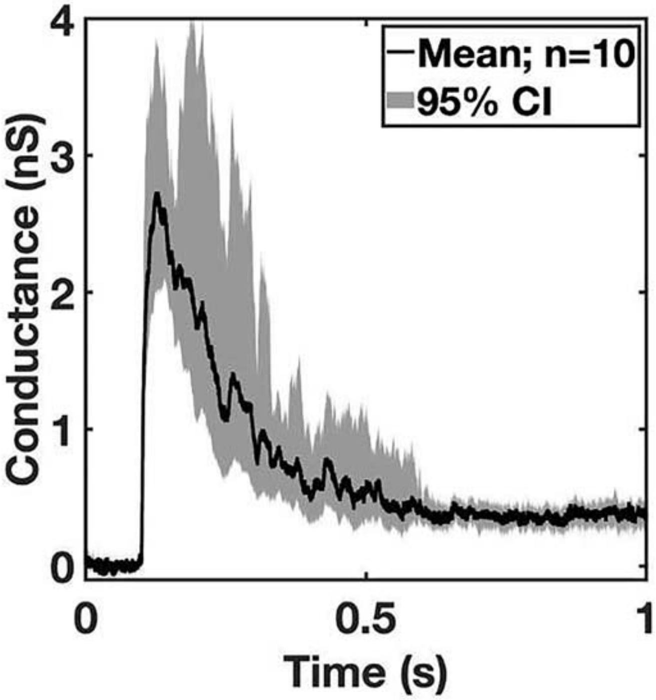

Figure EV4. Transients induced by WT parasites in low external calcium concentration (0.1 mM $CaCl_2$) have similar properties to WT transients induced in regular LCIS (2.0 mM $CaCl_2$; Fig. 3A).

The average waveform of low-calcium transients displays the characteristic fast rise to peak conductance and slower recovery to a new baseline ($n = 10$). The confidence interval (CI) is calculated for each point along the averaged transient.

This is a U.S. Government work and not under copyright protection in the US; foreign copyright protection may apply

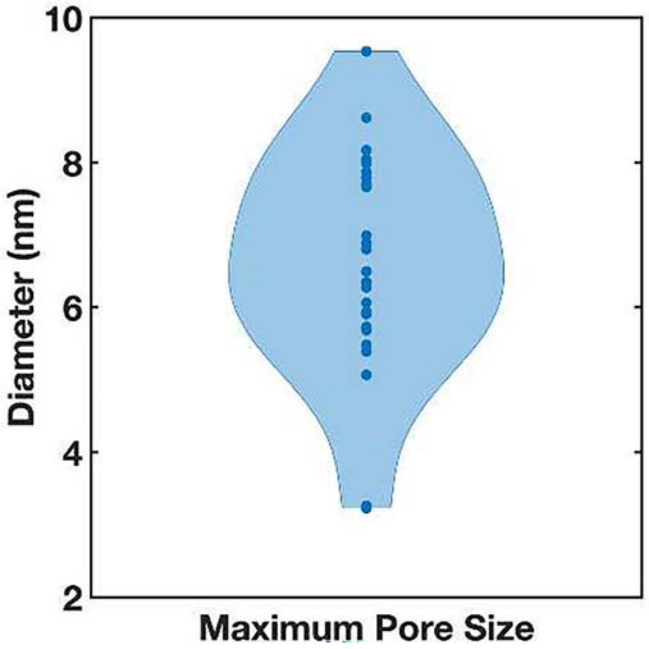

Figure EV5. Violin plot of pore diameters calculated from individual WT transient ($n = 25$) maximum conductance using a model for a cylindrical pore (see methods).

 This is a U.S. Government work and not under copyright protection in the US; foreign copyright protection may apply
